# Digital Mental Health Tools for Caregivers of Older Adults—A Scoping Review

**DOI:** 10.3389/fpubh.2020.00128

**Published:** 2020-04-28

**Authors:** Milica Petrovic, Andrea Gaggioli

**Affiliations:** ^1^Department of Psychology, Catholic University of Sacred Heart, Milan, Italy; ^2^Applied Technology for Neuro-Psychology Lab, Istituto Auxologico Italiano (IRCCS), Milan, Italy

**Keywords:** digital mental health interventions, caregivers, stress, burden, elderly care

## Abstract

**Aim:** Informal caregivers have an important role in bridging the gap between the assistance care recipients need and what can be provided by the health care systems across Europe. The burden of the caregiving role places a significant threat to caregiver health, and the vast majority of caregiver's report stress and emotional strain, depression, and increased rates of chronic diseases. In line with this, strengthening the caregiver's mental health is one of the main goals for optimal caregiving. Caregivers already struggle with the demand of their role while coping with health problems, social, family, and work obligations. The solution for the caregiver's mental health needs to be accessible, low cost, and time-effective. This scoping review investigates digital mental health tools available as a mean of supporting the mental health of caregivers.

**Method:** Databases searched include Summon search box, the Cochrane Library, and PubMed. Three groups of keywords were combined: relating to digital mental health interventions for caregivers, digital mental health interventions and stress in elderly care, and digital mental health interventions and burden in elderly care.

**Results:** Caregivers reported that digital mental health tools have an overall positive role in their health. Coping skills, emotion regulation, skill building, and education are found to be important aspects of digital mental health tools. There was a noted lack of digital mental health apps available specifically for the caregiver of older adults. Furthermore, the digital mental health tools, divided into three categories in this review, focused either on building skills or educating caregivers and assisting with the duties rather than the mental health of the caregiver itself. As repeatedly suggested in the reviewed studies, digital mental health interventions overall contribute to reducing the caregiver burden with a limitation of addressing one aspect of caregiver needs –i.e., specific coping skills or education regarding illnesses such as Alzheimer's disease and Dementia. The lack of all-encompassing, data and theory-driven digital mental health tools for addressing and supporting the caregiver's mental health is evident.

## Introduction

The rapid aging of the European population is one of the critical challenges the European social systems are facing today. Current predictions indicate that the number of individuals above 80 years of age will rise from 4.9% in 2016 to 13% in 2070 (1). The predicted rise puts social and health systems in Europe to a severe test and challenges the fiscal sustainability of long-term care while shedding the light on the current demographic changes. Family pattern changes, a higher number of single households, participation of women in the labor market, increased workforce mobility and an increase in retirement age are considered as important factors adding to the anticipated rise (1). Even though the existing health care delivery systems address some of the issues mentioned above, the care gap in the areas such as realigned reimbursement, team-based care, patient and family engagement, and information sharing still remain open (2).

Informal care has been generally defined as unpaid care provided for an older or dependent person with whom the caregiver has a close relationship such as spouse, parent, child, relative, friend or a close neighbor (3). The type of help provided by the informal caregiver varies based on the age, illness, and need of the care recipient and can include help with the household chores, running errands, providing transportation to the doctor, social and emotional support, distributing the medication, and providing physical care such as bathing and feeding (3, 4).

According to a study carried out by Piette et al. (5), care recipients with active and involved caregivers have better self-care and health outcomes than those with less involved caregivers. Moreover, those care recipients accompanied by the caregiver to the physician are more likely to discuss challenging and difficult topics related to their health and issues they are facing (5). Informal caregivers have an important role in bridging the gap between what health care systems can provide and the type of assistance and service the care recipient requires.

The burden of caregiving role includes the vast majority of difficulties reported by the caregivers and can be generally divided into physical, psychological and financial hardship (6). It is estimated that the informal caregivers spend on the average 24.4 h a week providing care, and this doubles to 44.6 h per week in cases where the care recipient is a spouse or a partner (7). The amount of care provided mostly depends on the care recipient—caregiver relationship—e.g., spouse, parent, sibling, friend—and the living arrangements between the two. Caregiving role comes with diverse challenges, and many of those put caregivers at risk of mental health problems (7–10) and even increased risk of mortality through the development of severe chronic conditions.

Primary sources of caregiver burden include lack of support network, not using formal and informal services for the caregivers, problem behaviors of the care recipients and insufficient or overwhelmed coping skills (11). Stress, depression, and burden overall lead to burnout, which deteriorates the quality of the caregiver's life and might also result in early institutionalization of the care recipient (12). However, the termination of caregiving does not end with the institutionalization of the care recipient into a nursing home. In fact, it has been noted that the responsibilities often increase since the attention at that point must be given to nursing home staff at ensuring they provide appropriate care in the absence of any family member (12). The caregiving role usually terminates when the care recipient has passed away. It has been suggested that the caregiving cycle might be repeated for the caregiver with another family member or relative (12).

The available interventions for informal caregivers fall into three main categories: respite, psychosocial interventions, and information and communication technology (ICT) support (13). Respite services provide the caregiver with a temporary break from the caregiving role and allow the caregiver time to rest and improve the well-being. The respite care, that temporarily overtakes the caregiving duties, has an overall positive impact on caregivers' burden after 2–3 months follow up (14). The psychosocial support interventions target the caregivers' ability to improve the management of caregiving situations. These interventions can be delivered either individually or as group support and are generally successful at providing caregivers with appropriate coping skills and strategies to deal with the demands of the role (15). The final category of interventions is ICT-based options for informal caregivers, such as digital educational platforms, and support services for stress, anxiety, and depression (16).

The ICT support provided for improving health, well-being, and health care are referred to as eHealth (17). Although terms such as eHealth, eHealth technology, eHealth interventions, health informatics, and behavior change interventions are used in the field of eHealth interventions, and often interchangeably. In this paper the term eHealth is used to refer to the digital support –i.e., mobile apps, web-based platforms, virtual reality, etc.—that delivers digital interventions or relevant educational content.

eHealth can enhance access to care, the empowerment of the patients and the healthy individuals, the innovation in health care and the new perspective on well-being. In order for an eHealth intervention to be considered successful, besides the theoretical aspect, behavioral modification background, and persuasiveness, it must be available regardless of the time and space, provide empowerment to people by allowing them more control of their healthcare, be a catalyst for innovation in healthcare and maintain the quality of care (17).

With respect to the numerous definitions and papers produced up to date, only a few colleagues (17, 18) provided a structured categorization of eHealth by offering three different categories:

- Categorizing eHealth technologies according to the *position they maintain in the healthcare continuum*
- Categorizing eHealth technologies according to the *characteristics of the technology*
- Categorizing eHealth technologies according to their *influence on the health-care systems*.

The eHealth categorization reveals how broad the field of eHealth is and the range of services and influences it can cover. However, for the purpose of this review the concept of eHealth is framed in terms of digital mental health tools, and narrowed down only to the digital tools—i.e., any technological and digital device used to distribute mental help interventions through mobile apps, web-based platforms, virtual reality, etc.—used to provide support and help manage health of the informal caregivers.

An increasing number of systematic reviews support the potential digital mental health tools hold for improving informal caregivers' well-being (19). Generally, it is suggested that web and smartphone-based interventions for caregiver populations may offer convenient, low-cost alternatives to visiting mental health professionals in weekly sessions or group settings. Technology-based interventions can be used at any time the caregiver is available. Furthermore, digital mental health tools can be personalized to address multiple issues that caregivers face on a day to day basis.

The purpose of this review is to investigate and thematically synthesize the existing literature, in order to understand the state of the art digital mental health tools for managing burden, stress, and overall adverse mental health outcomes for the informal caregivers. This scoping review is focusing on digital mental health tools available for the informal caregivers of older adults, with the important distinction of excluding caregivers of individuals with cancer. Namely, caregivers of people with cancer face challenges that can be distinguished from other caregiving roles (20). More specifically, they spend more time in their caregiving role, with care recipients experiencing more variability in symptoms and toxicities from different, multi-modal therapies which might lead to rapid health deterioration during a short period of time. In this sense, the caregivers of individuals with cancer are usually required to monitor the patient's health status frequently and in different ways than other caregivers, and use technical and psychosocial skills to promote care recipients' health (21). Therefore, digital mental health tools for the caregiver of an individual with cancer, unlike the digital mental health tools for the caregivers in general, must be tailored to provide a combination of specific skills set, coping skills and emotional regulation techniques.

This review does not individually assess the quality of the interventions used in each reviewed study, but rather explores and categorizes digital tools available to deliver the mental health interventions for informal caregivers. Moreover, another important note is that interventions for different health problems will not be explored since the variety of health problems experienced by caregivers ranges from physical to mental health problems and the number of interventions included would require broadening the research question and the aim of this review.

## Materials and Methods

### Data Sources and Search Strategy

**Figure 1:**
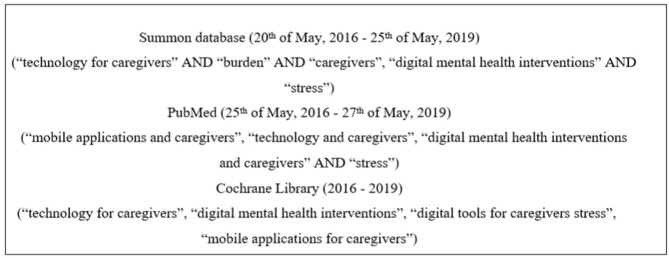
Outline of keyword searches used in the scoping review.

The electronic database Summon box (2016–2019) was searched to identify the existing reviews on the topic. The databases, including PubMed (2016–2019), and the Cochrane Library (2016–2019) were searched by combining three groups of keywords in all of the searches (Figure 1). The search focused on relatively new articles published in the last 3 years that contained keywords, for instance, “technology for caregivers AND burden,” or “digital mental health interventions AND caregivers” (see Figure 1). Retrieved articles were initially reviewed by the title and the abstract to find potentially relevant papers and exclude irrelevant ones. Relevant articles, that contained the keywords and clearly demonstrated in the abstract that the focus of the paper is on informal caregivers and digital tools available for them, were assessed against the inclusion criteria. Reference lists of relevant articles were reviewed to identify possible additional papers.

### Study Selection

#### Eligibility Criteria

We included (a) randomized controlled trials and observational studies which (b) investigated digital mental health tools available for informal caregivers or informal caregivers-care recipients dyads but offering support for the caregivers delivered via (c) mobile app, website or platform, tablet, virtual reality and mixed approach with web-based interventions combined with telehealth (d) aiming to reduced stress, burden, and adverse mental health outcomes and improve health and well-being of the informal caregiver. The trials and studies must have included (e) an adult population (≥18 years) with a preferable population (≥50 years) since the informal caregivers are statistically population above 50 years of age. We (f) focused on published peer-reviewed articles only, in English.

Digital mental health tools were defined as interventions and educational material provided for informal caregivers via mobile app, computers, tablets, virtual reality, and a mixture of tools such as mobile app and telehealth.

#### Inclusion Criteria

Selected papers were assessed against the following inclusion criteria:

(I) studies published in academic and peer-reviewed journals, (II) studies that are either quantitative or qualitative, (III) studies that answer “yes” to three screening questions, and (IV) studies published in English.

Screening questions:

1. Does the study address the use of digital mental health tools?
2. Does the study address digital mental health interventions?
3. Does the study include a caregiver—i.e., formal or informal—or digital mental health tool applicable to the caregiver adverse mental health outcomes –i.e., stress, burden, depression, and coping skills.

### Exclusion Criteria

The following categories of studies excluded:

- articles that did not address digital mental health tools;
- articles that did not include digital mental health content and digital mental health support;
- articles that included caregiver but maintained a focus on care recipient, without addressing the digital mental health tools for supporting caregiver's health;
- articles that included caregiver of children or young people only;
- articles including digital mental health tools for the caregiver of individuals with cancer;
- duplicate articles and articles not published in English.

### Data Extraction and Analysis

**Figure 2:**
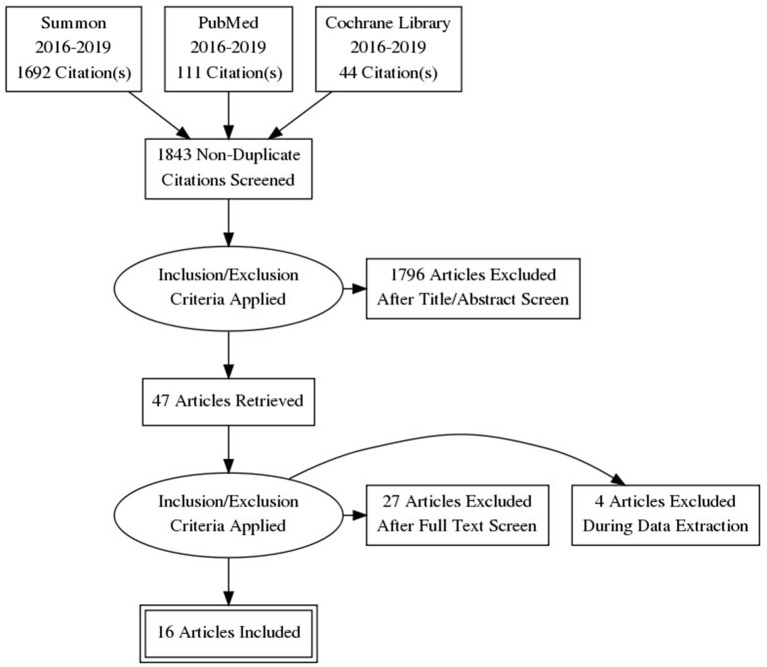
Flow of information through the different phases of a scoping review.

The screening of the titles and abstracts performed during the review (Figure 2) aimed to identify the studies that meet the inclusion criteria. Relevant studies were sorted and organized with the Zotero 4.0 software for further review. Full articles were reviewed to extract details about the study population, sample size, type of digital mental health tool, digital mental health intervention, and intervention outcomes (Table 1), conventional and novel findings.

**Table 1:** Summary table of studies included in the present scoping review.

**References**	**Population (sample size)**	**Intervention**	**Intervention outcomes**
Brown et al. (11)	Caregivers (*n =* 11), case-managers (*n =* 6), and primary care providers (*n =* 1). Mean age caregivers: 56.5 ± 13.5	CareHeroes: Web-based and Android application for caregivers of Alzheimer's and Dementia patients. Implemented in cross-institutional settings over 11 weeks period	50% of caregivers reported feeling more confident in determining solutions in new caregiving situations. 70% of caregivers found the application or web-based platform easy to use regardless of their primary knowledge of technology
Bush et al. (22)	Service veterans (*n =* 118). Mean age: 46 ± 13.5	Virtual Hope Box VHB: smartphone application for improving stress, coping skills, suicidal ideation and perceived reasons for living among patients with elevated risk of suicide	There was an overall decrease in stress and increase in coping skills. Users reported increase in ability to cope with unpleasant emotions and thoughts after using the application for 3-week period. The improvement remained stable entire trial period of 10 weeks
Callan et al. (8)	Primary caregivers (*n =* 27). Mean age: 74 ± 6.52	Adaptive Paced Visual Serial Attention Task (APVSAT): Computer-based cognitive training for the spousal caregivers of the individuals with Dementia	There was a noted increase in performance from the beginning till the end of the 4 week trial, with improve in problem-solving, coping, planning, and persevering with goal directed tasks
Croockston et al. (23)	Users of mental and emotional health applications (*n =* 150). The minimal age for participation: 18	Mindshift, Happify and other self-help applications: Association between theoretical behavioral change mechanisms and the use of self-help applications	Applications increased the overall motivation to be mentally and emotionally healthy. There was an increase in desire to set goals, maintain confidence and control
Frisbee et al. (24)	Caregiver-Veteran dyads (*n =* 882)	Care, RX Refill, Journal, Care4Caregivers, VA Pain Coach, VA PTSD Coach: Applications developed specifically for the study and available only for family caregivers of veterans who suffered severe physical and mental health injuries	Caregivers that experienced most burden, low preparedness, and high strain showed high usage of applications. The applications were used the most when the caregivers needed the assistance with the caregiving tasks
Grossman et al. (7)	200,000 mobile applications reviewed	iTunes, AppStore, and Google Play were searched. 44 applications were shortlisted to provide interventions for caregivers of older adults	36 applications generally addressed one of two categories: information and resources or, caregiver-care recipient interaction, while 8 applications addressed additional categories and provided stress reduction exercises
Ho et al. (9)	Informal caregivers (*n =* 20). Age range: 39–77	Mindfulness-based stress reduction MBSR: Aiming to improve psychological resilience of informal caregivers with a 8 weeks training course with the assessment of the blood gene expression profiles	Significant improvement in psychological resilience of some caregivers was reported. Predictive biomarkers were identified whose expression was associated with the greater benefit from MBSR training
Kajiyama et al. (10)	Latino/Hispanic caregivers (*n =* 25). Mean age: 63	Webnovela Mirela: Culturally adapted Spanish language series designed to educate and train caregivers to cope with care recipients with Dementia. The format was designed to be available without internet access with educational content adopted from “Active Caregiving Empowerment Skills”	There was a significant decrease in the levels of stress and symptoms of depression (*p* = 0.045)
Núñez-Naveira et al. (25)	Informal caregivers (*n =* 77), from Spain, Poland and Denmark	UnderstandAid: effectiveness of application for caregivers of people with Dementia	50% of participants evaluated positively technological and pedagogical specifications. There was a significant decrease of depressive symptoms
**References**	**Participants (sample size)**	**Intervention**	**Intervention outcomes**
Phongtankuel et al. (26)	Informal caregivers (*n =* 80). Mean age: 57 ± 12	mHealth: exploring the use of mHealth applications, caregivers receptivity and concerns	Informal caregivers reported the needs for: communication, caregiving information, education, updates from professional personnel, and scheduling services, as an important features for mHealth application
Piette et al. (5)	Heart-failure patients-Caregivers dyads (*n =* 396)	CarePartner: Systematic monitoring and interactive voice response calls about care recipients' health condition	Caregivers living away from care recipient who received CarePartner in combination with some mHealth reported lower caregiving strain even 12 months after the trial and significant improvement in depressive symptoms
Poonamallee et al. (27)	University students (*n =* 26)	DarmaLife program: smartphone application aiming to improve emotional intelligence by targeting maladaptive personality traits	DarmaLife had significant positive effect on emotional and social competency
Tam et al. (28)	Caregivers (*n =* 43). Mean age: 64 ± 16.41	Aging Service Technology AST: video educational program aiming to increase knowledge of caregivers about aging services	Younger caregivers (<65 years old) were more open to accepting the AST. Caregivers of the individuals who had fewer domains of functional limitation reported a positive change post-AST program
Tremont et al. (29)	Dementia care recipients and caregivers dyads (*n =* 250)	Telephone delivered interventions for caregivers—Family Intervention Telephone Tracking Caregiver FITT-C	The intervention FITT-C resulted in caregivers using community support services more and health resources less than caregivers in telephone delivered intervention with less Emergency department visits
Wijma et al. (30)	Informal caregivers (*n =* 42). Mean age: 55 ± 11.2	Virtual Reality intervention—Through D‘mentia Lens TDL: aiming to improve empathy in informal caregivers	TDL significantly improved empathy, confidence in caring and positive interactions between caregiver and care recipient
Zheng et al. (31)	Study1:Veterans Affairs MC VAMC (*n =* 155). Mean age: 67.78 ± 11.92. Study2: VAMC (*n =* 72). Mean age: 75.42 ± 9.49	Study1: Differences between computer-based and apps intervention usage. Study2: Differences between Telehealth devices and apps intervention usage	Group using computer-based interventions showed improvement in caregiving stress while Telehealth group did not

The reviewed studies were categorized according to the digital mental health tool used to deliver mental health intervention and support. Three categories included:

- Mobile apps
- Web-based
- Other digital tools.

The mobile apps category included all the relevant studies that addressed digital mental health support delivered via mobile-app for caregivers or apps addressing general adverse mental health symptoms relevant to caregivers. The web-based category gathered all the studies that included digital mental health support delivered to the caregiver in the form of web-based intervention, web-platform, with or without internet connection needed and web-based video programs or training. The final category of other digital mental health tools contains a study that reviewed virtual reality (VR) training for caregivers and telehealth content for caregiver-care recipient dyads combined with the data tracking by the master's level mental health practitioner.

The study result sections within each category were coded and thematically synthesized in order to gain a better perspective about the aim and purpose of each category and the method or intervention used to address the caregiver's adverse mental health symptoms. The categories were then compared and critically evaluated.

## Results

A total of 1,843 relevant non-duplicate records were identified. After applying exclusion criteria, 47 articles were retrieved eligible for full-text screening. Of those, 20 articles met the inclusion criteria, however, four were excluded during the data extraction due to the insufficient description of the study participants resulting in the unclear understanding if the study focused on the caregiver or digital mental health tool that can be used by the caregiver, vague description of procedure or limited report of the results. Out of 16 final articles with four RCT, two were mixed-methods, two were qualitative, and thirteen were quantitative studies. Summary of the results, including the authors, population, digital mental health tool or intervention, and the relevant intervention outcomes are provided in Table 1. The reviewed studies providing a comprehensive insight into the digital mental health tools available for the informal caregivers were distinguished by three categories.

The categories included: *mobile apps, web-based, and others*, and were determined by the device used to support the digital mental health content for the caregivers.

### Mobile Apps Category

The mobile apps category includes eight reviewed studies with the main focus on mobile-based mental health tools—i.e., mobile apps (7, 11, 22–27). The thematic analysis of the result sections within the mobile category indicated reappearing topics—e.g., *cope, stress, depression, self-regulation, behavior change, self-control*—that were further grouped into themes of “coping skills” and “emotional self-regulation.” This review suggests that the overall aim of the mobile apps category is centered on building coping and emotional regulation skills of a caregiver as means for dealing with the caregiving burden, stress and adverse emotions in general. The pre-post measurements available in three studies (22, 25, 27) indicated that the mobile apps helped decreased stress and increased coping skills after only 3 weeks, moreover, there was a reported significant positive impact on decreasing depressive symptoms of the caregivers and significant positive effect on emotional and social competency. The mobile apps category includes studies reflecting on apps that are designed for the caregivers specifically (e.g., iCare, RX Refill, Journal, Care4Caregivers, UnderstandAid, CareHeroes) and three mobile apps for dealing with adverse mental health symptoms in general, building emotional self-regulation, and the review of self-help apps -VA Pain Coach, VA PTSD Coach, DarmaLife Program, Mindify, Happify—(22, 23, 27).

### Web-Based Category

In the web-based category, six studies focused on web-based mental health tools (8–11, 28, 31). The thematic analysis of the result section of the studies indicated recurring topics—e.g., *education, information, exercising skills, resilience, training, coping, and distress*—merged into more general themes of “education” and “skill building.” The thematic synthesis indicated that the reviewed web-based digital mental health tools for caregivers aim to address caregiving burden and adverse mental health outcomes by educating caregivers about specific disorders and training proper skills for specific disorders necessary for the caregiving role. The web-based digital mental health tools were delivered in the form of video-educational programs, webnovela—i.e., intervention delivered through structured episodes in a form of small series similar to sitcoms—providing mindfulness-based stress reduction training via a web-based platform, and attention tasks via websites. One web-based mental health tool, CareHeroes, was an adapted web version of a previously reviewed tool in the mobile app category.

### Category of “Other” Digital Tools

The third category of “other” includes the final three studies exploring digital mental health tools that could not be classified as mobile apps or web-based mental health tools (5, 29, 30). The mental health tools in question are virtual reality intervention, telephone-delivered intervention for caregiver-care recipient dyads, and combined digital mental health intervention with “CarePartner” systematic monitoring with interactive voice response call for caregivers living outside of the care recipient home.

## Discussion

This review aimed to explore the current state of the art digital mental health tools for informal caregivers used as an intervention, support, means of education, and training, that could provide set of skills necessary for an individual to maintain health and well-being while fulfilling the role of a caregiver. The review seeks to understand means by which current digital mental health content available for the informal caregivers is delivered (e.g., via mobile phone, web page, tablet, virtual reality) and purpose these mental health tools are used for (e.g., intervention, training, education, support). The further exploration of the intervention was not performed since the scope of this paper is to review the tools used for distributing the mental health content related to the caregivers.

The thematic synthesis of the data indicated that the digital mental health tools currently available for caregivers could be broadly categorized as mobile apps, web-based, and other digital mental health tools—i.e., digital mental health tools that do not fit the first two categories. It is suggested that each category serves a different purpose.

- Mobile apps generally address adverse mental health by building skills, such as coping skills and emotional self-regulation, necessary for the caregivers to maintain well-being.
- The web-based mental health tools serve an overall purpose of educating and informing caregivers about the caregiving role and providing skill exercise and training for the caregivers.
- The third category of “other” digital mental health tools includes virtual reality intervention for the caregiver of individuals with Dementia, telephone-delivered intervention combined with digital mental health tool and mental health intervention with systematic monitoring and voice response designed for caregivers living away from the care recipients. This category includes digital mental health tools that address caregiver understanding of the care recipient and caregiver-care recipient communication, and although these digital mental health tools can be useful in improving caregiver's mental health by strengthening the caregiving role, they do not specifically address adverse mental health symptoms.

It can be suggested that the vast majority of mobile apps reviewed provide skill building for caregivers, while the web-based digital mental health tools provide skill training or skills exercising, and education, pointing out that one does not exclude the other, suggesting that they can be used jointly. Therefore, combining the digital tools can lead to creating a useful mixed tool for addressing the caregiver's mental health, burden, and stress at the more general level regardless of the limitations or illness of the care recipient. On the other hand, it can be argued that combining digital mental health tools can be rather overwhelming and burdening for the caregivers in terms of the information exposure and the time amount it would require from the caregiver.

The quality of the therapeutic basis of the mental health interventions provided through mobile apps reviewed in this paper has not been assessed. In fact, brief literature searches for papers assessing the therapeutic quality of the interventions provided through mobile apps has been rather scarce and often requires narrowing the search to a specific mental health issue. Some studies pointed out high-quality scores for mobile app interventions based on engagement, functionality, and aesthetics, as well as the potential to increase access to mental health services (32, 33). However, other studies simply provided guidelines and versions of protocols for assessing the quality of mobile app interventions for health care providers (34), without clear understanding about the protocols that need to be followed when creating mobile health apps in the first place (35).

The summary of the relevant studies in this review suggests general positive outcomes for caregivers after using the digital mental health tool with a reported increase in coping skills or emotional regulation (8, 10, 22, 27, 31), a decrease in stress, and burden (7, 22, 31) as well as perceived improvements in motivation to care (23). There were no adverse effects of digital mental health tools reported, even in the population that had low familiarity with technology, which might be further argued as a limitation. In line with this, the positive outcomes have been reported cross-sectionally in most studies with no longitudinal data available, pointing out that the positive outcomes might be only temporary.

Digital mental health tools were reported as useful for the caregivers that experienced higher burden and stress and were generally in more distress due to caregiving tasks (24). It has been shown that culturally-adapted digital tools are the right approach for educating caregivers, improving coping skills and motivation to care (10).

There was an evident lack of digital mental health tools aiming to support the mental health of the caregivers of older care recipients with overall 44 apps promoted on the market as such, while only eight of these addressed additional categories besides information and resources, communication and caregiver-recipient interaction (7). Furthermore, the majority of digital mental health tools are strongly focused on improving the caregiving role itself, which benefits care-recipients the most.

The digital tools available center on specific limitations or illnesses such as Dementia or Parkinson's disease, without offering the all-encompassing digital mental health tool for caregivers. Namely, a caregiver of an elderly individual without specific limitations or health issues might not benefit from a digital mental health tool designed for the caregiver of the individual with Alzheimer's disease and vice-versa. Most of the digital mental health tools cannot be applied in all-case scenarios. This leaves the caregiver of older people, with physical limitations but not specific mental health difficulty, outside of the research focus.

After using some of the digital mental health tools reviewed in this study, caregivers reported openness and interest in this type of technology (24), pointing out the potential interest by the caregivers to expand the knowledge and use the technology. The evident lack of digital interventions for caregivers goes in line with the lack of research data and literature exploring the specific issue. Current literature on caregiving is aiming to improve care recipients' well-being and explore the steps that can be taken by caregivers in order to further improve the quality of life and quality of care for the care recipients. Caregivers, on the other hand, seem to be the disregarded majority.

The reviewed studies reported that the caregivers, when asked to, were able to provide suggestions and describe the digital mental health technology they need in order to improve their well-being. This indicates that there is a clear lack of practical approach in creating digital mental health tools, content, and interventions, by simply involving caregivers in the creation of technology intended for them. In line with this, the development of digital mental health tools such as digital mental health intervention or generally positive technology for dealing with caregiver burden, besides scientific theoretical background, may also include caregiver suggestions and preferences since they have the most experience with caregiving and the mental health needs they develop over a course of role.

The main literature gap in this area of research is mostly centered around the lack of conclusive evidence and clear explanations regarding the effectiveness and therapeutic design in existing digital mental health tools addressing adverse mental health issues caregivers face throughout their role. Out of 16 reviewed papers, only a small portion provided clear the therapeutic rationale behind the interventions used. Furthermore, there is an evident gap in the population samples when it comes to the caregivers of older adults. Although informal caregivers of elderly individuals are a rather vast group, faced with similar difficulties of the caregiving role, it can be quite difficult to address numerous negative mental health issues with just one digital mental health tool. As noted during this review as well, studies generally focus on specific limitations or illness of the care recipient rarely including or directly addressing the caregiver. The noted lack of digital mental health interventions available for the caregivers of elderly individuals can also be one of the possible explanations for the literature gap in population samples earlier mentioned.

Even though numerous digital mental health interventions are available on the market, only a small portion can address adverse mental health effects of the caregiving role specifically. There is a grave need for the digital mental health tool designed for caregivers of elderly people that can cover a variety of needs caregivers experience in different stages of their role. In other words, a digital mental health tool for caregivers should provide caregivers with the deeper meaning-making and understanding of the caregiving role regardless if they joined the role voluntarily or were forced to due to the lack of alternative caregiver. A better understanding of the role effect on the caregiver's life and life adjustments that took place to accommodate the caregiving role must be taken into consideration. The effects of the role should be considered as an important factor that can provide insight and possibly be the predictor of the symptomatology hence could be utilized for the development of the preventive digital mental health tools and content.

The majority of mobile apps, web-based interventions, and other categories of digital mental health tools reviewed, for addressing caregiver stress, are based on the grounds of Cognitive Behavioral Therapy (CBT) or Stress Inoculation Training (SIT). In other words, the delivered digital interventions had a therapeutic background focused on readjusting cognitive patterns related to the adverse mental health effects of the caregiving role. In this way, the interventions were used to shift the emotional well-being of the caregivers or build up stress resilience by preparing the caregivers for adverse mental health effects of the caregiving role through skills training and educational material. The therapeutic background of several reviewed studies was not clearly defined and it reflects the general literature gap in the therapeutic rationale for digital mental health tools available for the caregivers.

It can be suggested that current digital mental health tools available for caregivers have been somewhat successful at targeting adverse mental health outcomes arising from the caregiving role with noted lack of structure, approach and therapeutic background of interventions. The necessity for a structured digital mental health tool for caregivers with clear theory, protocols, and frameworks is evident in cases where the caregiver is providing care for the recipient that has no specific mental or physical disability.

This scoping review is conducted in order to explore digital mental health tools available for informal caregivers. It is meant to add up to the efforts of other colleagues in filling in the literature gap in informal caregiving and digital mental health research. The results indicated several important points that can be used in further reviews, as well as important points that can guide future digital mental health tools development. These points include important aspects that were successfully included in the digital mental health tools reviewed: coping skills, emotional self-regulation, education, skill-building, and skill training.

In other words, caregivers benefited and showed improved well-being, lowered levels of stress and burden and increased emotional regulation after they were educated about the caregiving role, and they were given certain skills to manage their mental health and well-being while providing care. Teaching caregivers coping skills and emotional self-regulation is an important aspect of the reviewed digital mental health tools and can be argued as an important factor in the overall positive results achieved in the studies reviewed. Another important aspect of digital mental health tools noted in numerous studies reviewed is that they provided a space for caregivers to train or exercise the skills they have acquired, therefore obtaining good mastery over the skills taught. It can be suggested that this allowed caregivers to maintain certain resilience toward stressful events and overall role burden.

## Limitations

The lack of clear guidance about the efficacy of the mental health interventions delivered via digital tools explored in this review is considered the main limitation of this review. Namely, the majority of reviewed studies emphasized the effectiveness and success of the digital tool without reflecting on the therapeutic background or rationale for using a specific therapeutic approach for certain mental health issues. Although this review explored the usability of digital mental health tools among caregivers of older adults and the types of digital mental health tools currently available, it cannot provide any definitive conclusion about the efficacy of the interventions distributed through digital mental health tools as well as the caregivers' preferences when it comes to the type of digital mental health tools available. It could be the case that the interventions that had higher therapeutic potential were delivered in a way that was less convenient for the caregiver or the digital mental health tool used was not the best option for delivering the specific intervention.

Although several studies reported the success of digital mental health tools used, there was no clear checklist followed, and only one study provided follow-up results. Moreover, the comparison between the digital mental health tools available has not been made in any papers, therefore it cannot definitively be concluded that one digital mental health tool or a specific type of technology is more successful than the other. For instance, the educational intervention in a form of Webnovela “Mirella” for Hispanic caregivers was rather successful at providing a set of skills that caregivers reported as useful in their caregiving role. However, there is no clear indication if this intervention could be equally, more, or less successful when delivered via different digital tools such as mobile phones, tablets, web, or even as a VR experience. Finally, the number of papers exploring the digital mental health tools for caregivers is still quite limited which prevents a clear insight into the topic.

Despite its limitations, this review noted numerous positive aspects and suggests that digital mental health tools can be an inexpensive, easily accessible, and time-saving option for addressing the caregiver burden and mental health. There is a need for further improvements and development of commercialized digital mental health tools that will be science-based but caregiver tailored. Moreover, it can be suggested that further development should include pilot testing over different platforms and by using different tools in order to establish the optimal digital tool for each mental health intervention or caregiver-tailored content.

Moreover, according to the results obtained in this review, digital mental health tools for caregivers that provide coping skills, emotional self-regulation skills, education about caregiving, skill-building and skill training in a well-structured approach are the most successful in managing caregiver stress and burden.

Finally, digital mental health tools, including web-based, mobile apps, or virtual reality solutions, have the potential to reshape health care due to its ability to be structured in a therapeutic way, providing interventions for a wide variety of caregivers regardless of their age and personality characteristics, through video, audio, text and interactive content. Due to its affordability, accessibility, adaptability, and ability to deliver structured and therapeutically based interventions, digital mental health tools can be considered as potential next step support for informal caregivers.

## Author Contributions

MP wrote the manuscript with support and under the supervision of AG. MP wrote the main manuscript while AG participated in every aspect of it with guidance, additional paragraphs, and numerous corrections.

## Conflict of Interest

The authors declare that the research was conducted in the absence of any commercial or financial relationships that could be construed as a potential conflict of interest.
